# Measurement agreement between a newly developed sensing insole and traditional laboratory-based method for footstrike pattern detection in runners

**DOI:** 10.1371/journal.pone.0175724

**Published:** 2017-06-09

**Authors:** Roy T. H. Cheung, Winko W. An, Ivan P. H. Au, Janet H. Zhang, Zoe Y. S. Chan, Alfred Man, Fannie O. Y. Lau, Melody K. Y. Lam, K. K. Lau, C. Y. Leung, N. W. Tsang, Louis K. Y. Sze, Gilbert W. K. Lam

**Affiliations:** 1 Gait & Motion Analysis Laboratory, Department of Rehabilitation Sciences, The Hong Kong Polytechnic University, Hong Kong, China; 2 Persona Surgical Modelling, Hong Kong, China; 3 Li Ning Sports Science Research Center, Beijing, China; Northwestern University, UNITED STATES

## Abstract

This study introduced a novel but simple method to continuously measure footstrike patterns in runners using inexpensive force sensors. Two force sensing resistors were firmly affixed at the heel and second toe of both insoles to collect the time signal of foot contact. A total of 109 healthy young adults (42 males and 67 females) were recruited in this study. They ran on an instrumented treadmill at 0°, +10°, and -10° inclinations and attempted rearfoot, midfoot, and forefoot landings using real time visual biofeedback. Intra-step strike index and onset time difference between two force sensors were measured and analyzed with univariate linear regression. We analyzed 25,655 footfalls and found that onset time difference between two sensors explained 80–84% of variation in the prediction model of strike index (R-squared = 0.799–0.836, *p*<0.001). However, the time windows to detect footstrike patterns on different surface inclinations were not consistent. These findings may allow laboratory-based gait retraining to be implemented in natural running environments to aid in both injury prevention and performance enhancement.

## Introduction

There has been an increasing interest in footstrike pattern detection during running, possibly because of its potential relationship with injury [1–3] and performance [4]. There are three types of footstrike patterns: rearfoot strike (RFS) refers to when the heel lands before the ball of the foot; forefoot strike (FFS) refers to when the ball of the foot lands before the heel; and midfoot strike (MFS) refers to when the ball and heel of the foot land at similar times [5]. It has been reported that RFS, when compared with MFS or FFS, results in a higher impact loading [6], which has been associated with many running injuries [7]. However, greater calf strain may result from a FFS pattern, which may cause Achilles tendinitis [8]. Biomechanically, a MFS is the optimal footstrike pattern but past research has indicated that runners find it difficult to self-adjust to a MFS [1]. Regarding running performance, when speed is increased by 1 m/s, the odds of having a FFS or MFS relative to RFS increased by 2.3 and 2.6 times, respectively [9]. Hence, it may be beneficial to provide real time information about footstrike pattern to runners.

Currently, measurement of footstrike pattern in consecutive footfalls is mainly restricted to a laboratory setting. Footstrike pattern is usually determined kinetically by the strike index (SI), which measures the location of the center of pressure (COP) at initial contact along the long axis of the foot as a percentage of the total foot length [10]. A RFS, MFS, or FFS is defined as such when the SI is between 0–33%, 34–66%, and 67–100%, respectively. Unfortunately, force data are not always available during continuous running. In addition, un-optimized signal-to-noise ratio at initial contact may affect the accuracy of the COP calculation [11]. More importantly, SI measurement requires sophisticated equipment and therefore continuous footstrike pattern detection outside a laboratory environment remains difficult or costly.

With the advancement of sensor technology, emerging studies have explored the potential application of wearable sensors for gait analysis [12,13], which would allow for outdoor biomechanical measurement. In terms of footstrike pattern detection, a previous case series used a simple circuit, which consisted of a single force sensing resistor at the heel region, to modify footstrike patterns in runners [14]. However, in that particular study, the device appeared to be unable to differentiate between MFS and FFS. In view of the fact that footstrike pattern refers to the timing and the location of foot at contact, an additional force sensing resistor at the toe region may be a potential solution for footstrike pattern detection. More specifically, it is expected that a RFS may trigger the heel sensor before the toe sensor, and vice versa for FFS; while MFS may trigger both sensors at similar times. As a result, the onset time difference (OTD) between heel and toe sensors may provide a surrogate measure of SI. We believe that a mobile measurement of footstrike pattern may prevent injury and enhance running performance by gait retraining, which is an intervention shown to effectively modify running biomechanics [15].

Hence, this study sought to determine if SI can be estimated by measuring the OTD between heel and toe sensors across various surface inclinations. We hypothesized that as OTD increased (towards earlier onset of toe sensor relative to the heel sensor), SI would decrease across the full spectrum of footstrike patterns.

## Materials and methods

### Subjects and procedures

A total of 109 healthy adults (42 males and 67 females; age range = 18–38 years; mean age = 21.5±4.2 years; height = 1.67±0.09 m; mass = 56.8±8.3 kg) free from any active lower-extremity injuries were recruited. All of the subjects signed an informed consent form and the experiment was approved by the Departmental Research Committee of the Department of Rehabilitation Sciences, The Hong Kong Polytechnic University.

**Fig 1:**
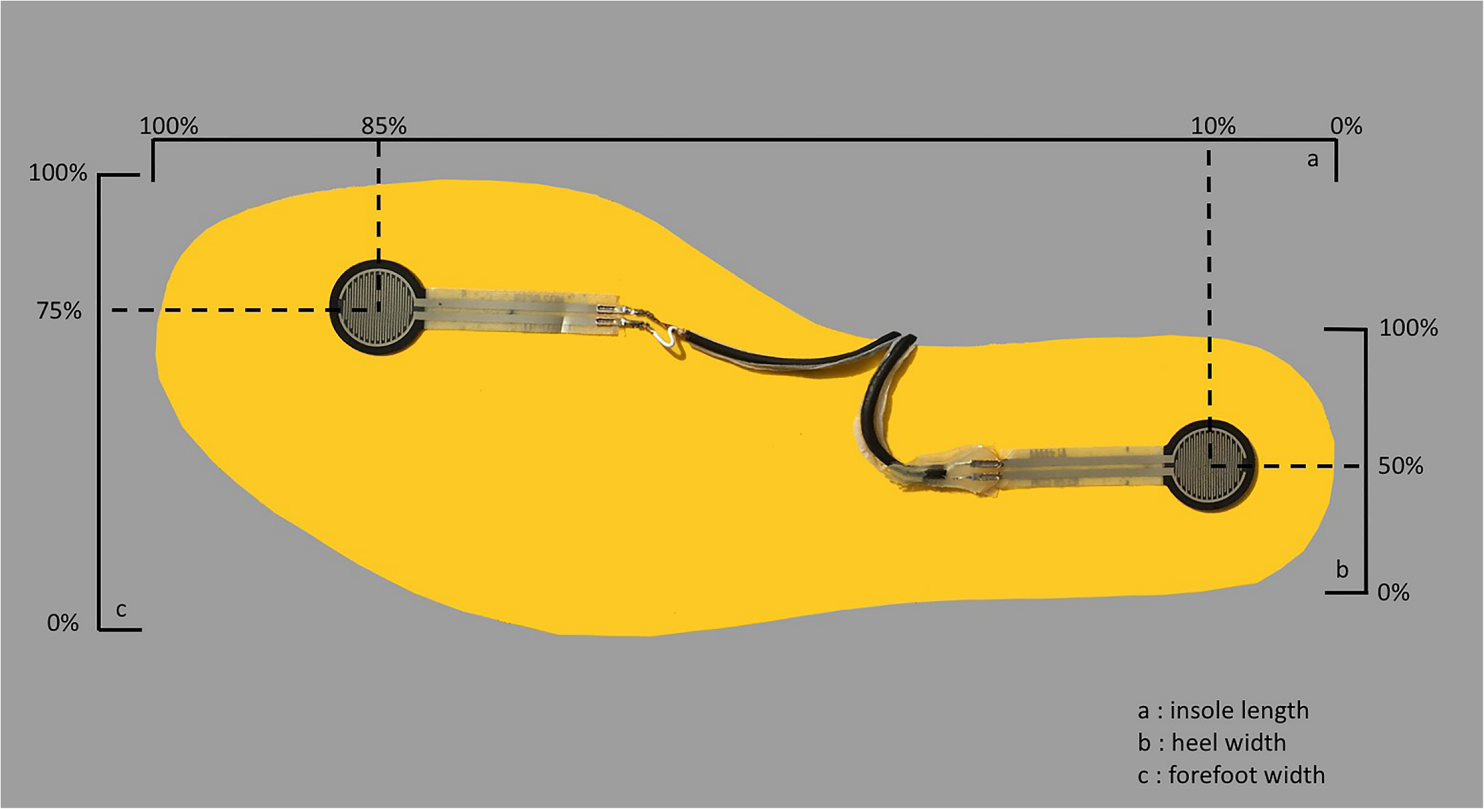
Location of the force sensing resistors.

Two force sensing resistors (1087-1001-ND, Digi-Key, MN, USA) were firmly affixed at the heel and the second toe of both left and right insoles to collect the time signal of foot contact (Fig 1). Reflective markers were placed according to a previously established model [11]. After standardized lower limb stretching exercise as a warm-up, each subject was asked to run on a self-paced instrumented treadmill (AMTI, Watertown, MA, USA) with the standard test shoes (ARHK, Li Ning, Beijing, China) at 0°, +10°, and -10° inclinations. All of the subjects were asked to RFS, MFS, and FFS with an augmented visual feedback [16]. The trial sequence (3 inclinations x 3 footstrike patterns) was randomized using an online program (www.randomizer.org). Each running condition lasted for two minutes with a 1-minute rest interval to avoid fatigue [17].

### Data analysis

Ground reaction force and motion data were recorded at 1,000 and 200 Hz respectively before being filtered at 50 Hz with a fourth order Butterworth lowpass filter [18]. The last 30 footfalls in each running trial were extracted for analyses. The SI of each footfall was computed according to the method described in a previous study [10]. Corresponding OTD was also measured and was defined by the triggering time of the toe sensor relative to the heel sensor. A negative OTD thereby indicated an earlier onset of the heel sensor compared to the toe sensor and vice versa. In order to compare subjects with different foot sizes, the OTD was normalized with subjects’ foot length. Specifically, the measured OTD was linearly corrected by multiplying a ratio between the subject’s foot size and a standard length (23 cm).

### Statistical analysis

Univariate linear regression was performed to assess intra-step agreements between OTD and SI. We also conducted subgroup analyses according to different surface inclinations. All data was processed using SPSS.21^®^ statistics software of package (Chicago, IL, USA). Global alpha was set at 0.05.

## Results and discussion

A total of 29,430 footfalls (109 subjects x 3 inclinations x 3 footstrike patterns x 30 footfalls) were collected. However, 3,775 steps were excluded as the COP of their initial contact were at the split portion of the treadmill and therefore, a total of 25,655 footfalls were analyzed (S1 Table). The relationships between SI and OTD is shown in Table 1.

**Table 1:** Linear univariate regression analyses predicting strike index (SI) from onset time difference between heel and toe sensors.

							95% CI of B	
	Surface	B	t	Beta	VIF	*p*	Lower Bound	Upper Bound
SI	All	0.444	268.69	0.859	1.0	<0.001	0.441	0.448
	Flat	0.440	215.77	0.914	1.0	<0.001	0.436	0.444
	Inclined	0.425	179.10	0.894	1.0	<0.001	0.420	0.430
	Declined	0.507	205.43	0.913	1.0	<0.001	0.502	0.512

The linear regression models with SI across surface inclinations are shown in Eqs (1–4) below.

$$\mathrm{Overall}:\;SI=0.444*OTD+45.84,R^{2}=0.836$$ (1)

$$\mathrm{Level}:\;SI=0.440*OTD+42.27,R^{2}=0.836$$ (2)

$$\mathrm{Inclined}:\;SI=0.425*OTD+57.02,R^{2}=0.799$$ (3)

$$\mathrm{Declined}:\;SI=0.507*OTD+39.00,R^{2}=0.833$$ (4)

With the Eqs (1–4), corresponding OTDs at 33% and 66% SI were computed (Table 2).

**Table 2:** Cutoff onset time differences (ms) at 33% and 66% strike index (SI).

		95% CI			95% CI	
Surface	SI at 33%	Lower Bound	Upper Bound	SI at 66%	Lower Bound	Upper Bound
All	-28.92	-29.12	-28.66	45.41	45.71	45.00
Flat	-21.07	-21.26	-20.88	53.93	54.43	53.45
Inclined	-56.52	-57.19	-55.86	21.13	21.38	20.88
Declined	-11.83	-11.95	-11.72	53.25	53.78	52.73

This study described a sensing insole design including a simple circuit to predict footstrike pattern in distance runners. The data showed that OTD explained 80–84% of the variation in the prediction model of SI. In view of the response time (<3 ms) of the sensor used in this study, the time windows should be sufficient to differentiate between RFS, MFS, and FFS in distance runners.

Such technology could potentially be used to build a sensing insole to continuously measure footstrike patterns in runners. However, we found that the OTDs to detect footstrike patterns during running on surfaces at different inclinations were not consistent. Future application of our findings should consider the surface inclination using other devices e.g. gyroscope. We acknowledge that there are currently other pressure sensing insoles available on the market, such as Pedar (Novel, Munich, Germany) or F-scan (Tekscan, Boston, MA, USA) systems, which can fulfill the same purpose. However, the current system, which contains two force sensing resistors and costs approximately US$20, should be more cost effective than the existing pressure sensing insoles. Some researchers have used a foot-mounted accelerometer to detect footstrike pattern in runners [19]. However, such measurements may disregard the fact that some runners with MFS or FFS may still present with high impact loading [20] and, therefore, could lead to the misclassification of footstrike patterns. In view of its cost (US$20) and measurement accuracy (R squared up to 0.84), the proposed device should be regarded as a cost-effective method to continuously measure footstrike patterns in runners.

Laboratory-based gait retraining has been used to prevent running injuries, rehabilitate injured runners, and enhance running performance [21,22]. For instance, a previous laboratory-based gait retraining program sucessfully modified footstrike pattern in a group of runners with patellofemoral pain leading to an improvement in runners’ symptoms after training [18]. However, laboratory accessibility is usually limited and subject preparation for a biomechanics evaluation or gait retraining is time consuming and resource driven. More importantly, whether or not the subjects are able to maintain the newly learnt motor pattern outside of the laboratory is questionable. Therefore, the findings of the present study may be able to allow gait retraining outside the laboratory environment.

We have several limitations in the present study. Firstly, we did not test the sensing insole in other shoe models. This test shoe was selected because it is a neutral shoe model with nominal foot arch support. Such a design allows for a flat shoe-foot interface and avoids mistriggering due to insole torsion. Secondly, we recruited only young healthy adults for this study, which may limit the generalization of our findings to the elderly or injuried runners. Thirdly, the relationship between OTD and surface inclination was not explored at extreme inclination angles and future study in this area is therefore warranted. Finally, the prediction model may be further enhanced by considering other parameters, such as running speed.

## Conclusions

In the present study, we describe a novel but simple method to continously measure footstrike patterns in distance runners using inexpensive sensors.

## Supporting information

S1 Table Strike index and corresponding OTD when running on different surfaces. (XLSX)
